# Quantitative Trait Loci Mapping and Identification of Candidate Genes Linked to Fruit Acidity in Apricot (*Prunus armeniaca* L.)

**DOI:** 10.3389/fpls.2022.838370

**Published:** 2022-03-18

**Authors:** Luca Dondini, Cecilia Domenichini, Yonghui Dong, Fabio Gennari, Daniele Bassi, Stefano Foschi, Martina Lama, Marco Adami, Paolo De Franceschi, Claudia Cervellati, Lorenzo Bergonzoni, Sara Alessandri, Stefano Tartarini

**Affiliations:** ^1^Department of Agricultural and Food Sciences, Alma Mater Studiorum – University of Bologna, Bologna, Italy; ^2^Department of Agricultural and Environmental Sciences (DISAA), University of Milan, Milan, Italy; ^3^Rinova, Cesena, Italy; ^4^Astra Innovazione e Sviluppo, Imola, Italy

**Keywords:** fruit pH, malate, citrate, quinate, fruit quality

## Abstract

Apricot breeding programs could be strongly improved by the availability of molecular markers linked to the main fruit quality traits. Fruit acidity is one of the key factors in consumer acceptance, but despite its importance, the molecular bases of this trait are still poorly understood. In order to increase the genetic knowledge on the fruit acidity, an F1 apricot population (‘Lito’ × ‘BO81604311’) has been phenotyped for titratable acidity and juice pH for the three following years. In addition, the contents of the main organic acids of the juice (malate, citrate, and quinate) were also evaluated. A Gaussian distribution was observed for most of the traits in this progeny, confirming their quantitative inheritance. An available simple sequence repeat (SSR)-based molecular map, implemented with new markers in specific genomic regions, was used to perform a quantitative trait loci (QTL) analysis. The molecular map was also anchored to the recently published apricot genome sequence of ‘Stella.’ Several major QTLs linked to fruit acidity-related traits have been identified both in the ‘Lito’ (no. 21) and ‘BO81604311’ (no. 13), distributed in five linkage groups (LG 4, 5, 6, 7, and 8). Some of these QTLs show good stability between years and their linked markers were used to identify candidate genes in specific QTLs genomic regions.

## Introduction

Apricot (*Prunus armeniaca* L.) belongs to the Rosaceae family as well as several other valuable fruit tree species including peach, sweet and sour cherry, almond, Japanese and European plums. With a world total production of 4.08 million of tons in 2019 (FAOSTAT)^1^, apricot is the third most important stone fruit species in economic terms, after peach and plum. In the last 20 years, the apricot world production is almost doubled and this was possible by a remarkable increase in new plantations that occurred from 1991 to 2008.

Several pomological attributes define apricot fruit quality, including sensory properties (size, flesh firmness, color, texture, taste, and aroma), nutritional values, chemical compounds, mechanical properties, and functional properties. These attributes are significant for the global market and for consumers who, in the last decades, steered apricot breeding to increase some fruit traits such as firmness and blush color and also fruit taste (Tricon et al., 2009). Fruit quality plays a fundamental role in the acceptance of apricot cultivars by consumers; therefore, improving fruit quality is one of the main objectives in apricot breeding programs.

Acidity is one of the factors that most affects taste and for this reason it is a primary quality attribute of the fruit. In general, the acidity of the fruit flesh is given by the intracellular accumulation of organic acids and specifically by the balance between their synthesis, degradation and vacuolar storage (Etienne et al., 2013). Malate, citrate and quinate are the predominant organic acids in apricot fruits and they are involved in numerous metabolic processes that determine their cell concentrations, including the tricarboxylic acid (TCA) cycle, the mitochondrial energy metabolism, the glyoxylate cycle, the γ-aminobutyrate (GABA) shunt and acetyl-CoA catabolism (Xi et al., 2016). In all these processes, the conversions of organic acids are mediated by specific enzymes. Concerning malate, the first major organic acid in apricot, malate synthase is the key enzyme for its synthesis. Despite the lower content, in respect to malate, citrate has a strong impact on the global taste being the sourest organic acid in the fruit. Its accumulation is because of the citrate synthase activity within the TCA cycle, and in a lesser extent to glutamate decarboxylase (GAD) activity in the GABA shunt pathway. Quinate is produced by quinate dehydrogenase, an enzyme participating in phenylalanine, tyrosine and tryptophan biosynthesis (Xi et al., 2016). These three organic acids show a decrease in the last stages of fruit ripening that may be explained by a reduction of the synthesis of the enzymes involved in their production and an increase in those involved in their degradation, i.e., phosphoenolpyruvate carboxylase, NADP-malic enzyme and the NAD-malic enzyme for malate (García-Gómez et al., 2019).

As high concentrations of organic acids can inhibit the activity of the respective synthesis enzymes, the accumulation of these compounds in fruits strongly relies on their sequestration into vacuoles. Therefore, another important class of proteins affecting fruit acidity are vacuolar transporters. In apple, an aluminum-activated malate transporter was identified as the main responsible of fruit malate content (Bai et al., 2012).

Malate and citrate cross the tonoplast in the dianion and trianion forms, respectively, by facilitated diffusion through the same channels (Oleski et al., 1987; Rentsch and Martinoia, 1991). However, citrate is favored over malate owing to the vacuolar pH and the electrochemical potential gradient across the tonoplast (Gout et al., 1993), but its accumulation in the vacuole is slower because it is controlled by its cytosolic concentration and thus by respiration (Hafke et al., 2003).

Metabolism and accumulation of organic acids are strongly influenced by both the genetic and environmental factors (Etienne et al., 2013). Regarding the genetic component, several works suggest that, like most other apricot fruit quality traits, the control of fruit acidity is polygenic and quantitatively inherited (Couranjou, 1995; Bassi et al., 1996; Signoret et al., 2004; Ruiz and Egea, 2008; Ruiz et al., 2010; Salazar et al., 2013, 2014, 2016; García-Gómez et al., 2021). The identification of genomic regions or quantitative traits loci (QTLs) involved in fruit quality traits is; therefore, a key step in improving traditional breeding programs and developing marker-assisted selection (MAS) protocols.

Various QTLs linked to fruit acidity have been described in different apricot progenies. Ruiz et al. (2010) found four QTLs in LG2, LG3, LG6, and LG7 in the ‘Goldrich’ × ‘Moniqui’ population. Furthermore, a preliminary QTL analysis on the ‘Lito’ × ‘BO81604311’ progeny highlighted the other three QTL in LG6, LG7, and LG8 (Ruiz et al., 2010). Salazar et al. (2013) observed some QTLs related to pH and malate content in LG1, LG2, and LG4 in a ‘Z701-1’ × ‘Palestine’ progeny. Recently, García-Gómez et al. (2019) placed emphasis on QTLs related to malic acid content on LG2 and LG8 of the ‘Goldrich’ × ‘Currot’ and ‘Bergeron’ × ‘Currot’ populations. The alignment of QTLs identified in different progenies is hampered by the lack of common markers among the different maps.

The identification of candidate genes in specific genomic regions is strongly facilitated by the availability of an apricot whole genome sequence. Recently, to this extent, two *Prunus armeniaca* high-quality assemblies were reported; the ‘Marouch’ and ‘Stella’ assemblies were then organized into eight pseudo-chromosomes using a set of 458 previously published molecular markers (Groppi et al., 2021).

The objective of this article is to identify candidate genes within the QTL regions for the major components involved in apricot fruit acidity (fruit pH, titratable acidity, and the main organic acids contents). The two available linkage maps of the ‘Lito’ × ‘BO81604311’ progeny, implemented in specific genomic regions were used for QTL analysis and for anchoring the QTLs to the new *Prunus armeniaca* ‘Stella’ genome (Groppi et al., 2021).

## Materials and Methods

### Plant Material

The plant material evaluated was the F1 apricot progeny derived from the cross between ‘Lito’ × ‘BO81604311’ (L×B), established at the Rinova (Cesena, Italy) and composed of 118 individuals. ‘Lito’ is a Greek cultivar derived from the cross ‘Stark Early Orange’ × ‘Tirynthos’ and it is known to be resistant to plum pox virus (PPV). ‘BO81604311’ is a breeding line from a cross between the two Italian cultivars ‘San Castrese’ × ‘Reale di Imola.’

### Phenotypic Evaluation

Samples of mature fruits for each seedling were hand-harvested and analyzed for titratable acidity (TA) and fruit pH over three consecutive years. The citrate, malate, and quinate contents were quantified only in the first and second year. Only in the first year, a number of representative fruits of the two parents were available for phenotyping.

Titratable acidity and pH were determined by an automatic titration system (Crimson Compact Titrator) on samples of 10 ml of juice from three mature fruits from each parent and seedling, then diluted with the same amount of water and titrated with 0.25 mol L^–1^ NaOH to pH 8.1.

The organic acids content (malate, citrate, and quinate) was measured by gas chromatography (GC) using the procedure described by Bartolozzi et al. (1997). Samples of 5 g of fruit flesh without peel were finely ground by an Ultra-Turrax homogenizer and made up to volume in 50 ml of buffer. An aliquot of each derivatized sample (1 μl) was injected into the GC column. Then, GC data were converted in mg/100 g of fresh weight.

### Data Statistical Analysis

Datasets of the phenotypical trait were analyzed by ANOVA and a *post hoc* Tukey’s test under R environment using ‘‘agricolae’’ R package^2^. A correlation analysis between the data of acidity’s component was carried out with ‘‘Multcomp’’ R package using Pearson’s method^3^. Additionally, the Shapiro–Wilk test was performed for the normality test.

### Deoxyribonucleic Acid Extraction, Markers Analysis and Genetic Linkage, and Physical Maps Construction

The mainframe of the SSR genetic linkage maps of ‘Lito’ and ‘BO81604311’ were already published (Dondini et al., 2007, 2011). This map was enriched with four new markers in order to close gaps mainly present in LG8. In particular, two new microsatellites (SSRs) and two single nucleotide polymorphisms (SNPs) were designed to cover the two bare regions of this LG spanning about 3.6 Mb: the first between UDAp-438 and UDAp-470 and the second between BPPCT012 and AMPA111. The new SSRs were identified on the peach genome sequence (Verde et al., 2013, 2017). The two SNP markers were identified by sequencing PCR fragments from the other two genes. Primers for each marker were designed using Primer3 (Supplementary Table 1). All the markers were designed within gene-coding regions.

Total genomic DNA from each sample was isolated from 5 mg of ground lyophilized leaves using the procedure described by Mercado et al. (1999) and later modified by Dondini et al. (2007). Quantification of dsDNAs was accomplished with NanoDrop*™* ND-1000 Spectrophotometer (Thermo Fisher Scientific, Wilmington, DE, United States) and samples were diluted at 10 ng/μl.

Extracted DNAs were amplified using a 2720 Thermal Cycler (Applied Biosystems). The PCR reaction for SSR markers was performed as described by Dondini et al. (2007). Amplified PCR products were separated by electrophoresis using 5% polyacrylamide gel stained with silver nitrate. The SNP markers were amplified trough Temperature Switch PCR (Tabone et al., 2009) and separated by electrophoresis using 1% agarose gel (1X SB buffer) stained with GelRed*™* Nucleic Acid Gel Stain^®^. The details of the amplification protocols are given in Supplementary Table 2.

Molecular maps of each parent were constructed with JoinMap 4.1 software (Van Ooijen, 2006). Markers were first grouped using a minimum LOD score of 5. Regression mapping by Kosambi’s function was used for calculating map distances.

The physical position of 240 mapped markers was searched on the available *Prunus armeniaca* ‘Stella’ genome sequence (Groppi et al., 2021). Forward and reverse primers were aligned with the reference sequence using the Basic Local Alignment Search Tool (BLAST) of the Genome Database for Rosaceae (GDR)^4^.

Linkage and physical maps were drawn using MapChart 2.0 (Voorrips, 2002).

### Quantitative Trait Loci Analysis and Candidate Genes Identification

Quantitative trait loci identification for characters related to the fruit acidity was performed using the software MapQTL version 6.0 (Van Ooijen, 2000) with composite Interval Mapping. The Kruskal–Wallis non-parametric test was also applied to confirm the QTL identified by Interval mapping, especially for traits with non-normal distribution in the progeny (Alonso-Blanco et al., 2006). The Kruskal–Wallis test data were also used for better defining the QTL regions. Finally, a “permutation test” (1,000 permutations) was carried out to establish the genome-wide LOD score thresholds (corresponding to a probability higher than 95 and 99%, respectively) for each QTL. A QTL was considered as major when the percentage of explained variability was higher than 10%.

Putative candidate genes for acidity-related traits were searched at genome level by screening the predicted gene database of the *Prunus armeniaca* ‘Stella’ genome available on the GDR website^5^. In order to investigate the different QTL regions, the slope change in *K*-value, from the Kruskal–Wallis test, was used to delimit the QTL peaks where to look for candidate genes.

## Results

### Phenotypic Analysis

Fruits of ‘Lito’ resulted more acidic and with a lower pH in respect to ‘BO81604311’ (16.2 vs. 8.2 mg/l malic acid; 3.4 vs. 4.6 pH). The number of individuals tested in each year was slightly different because only seedlings that were able to provide a sufficient number of fruits for the analyses have been harvested.

**FIGURE 1:**
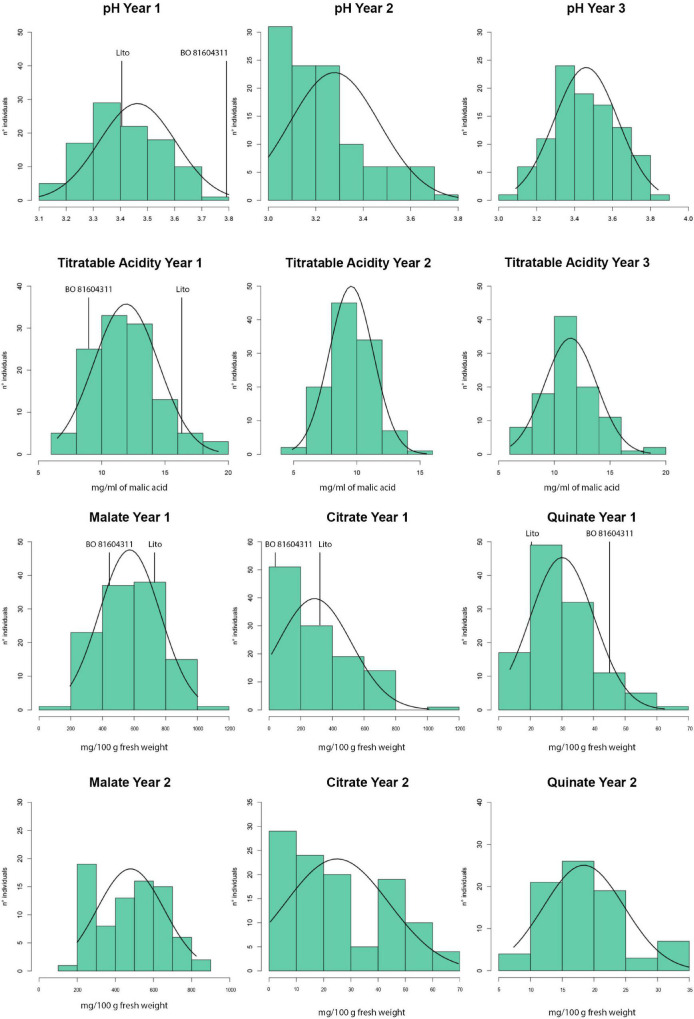
Distributions of the phenotypic traits among the progeny of ‘Lito’ × ‘BO81604311’ in the different years of analysis.

The titratable acidity and pH segregated in the progeny (Table 1) and a normal distribution was observed for several of the analyzed traits (Figure 1), as corroborated by the Shapiro–Wilk normality test (Supplementary Table 3). Exceptions have been observed for citrate and quinate contents in both the years.

**TABLE 1:** Mean, minimum, maximum, and SE of the traits observed in the F1 population and parents subdivided by year of observation.

Trait	Year	*n*°	F1				BO81604311				Lito			
			Mean	SE	Min	Max	Mean	SE	Min	Max	Mean	SE	Min	Max
pH	1	114	3.62a	±0.51	3.1	5.7	4.6				3.4			
	2	108	3.28c	±0.19	3.0	3.8								
	3	101	3.46b	±0.17	2.9	3.8								
Titratable acidity	1	116	12.02a	±2.76	6.5	23.2	8.2				16.2			
	2	109	9.55b	±1.74	4.9	15.5								
	3	101	11.46a	±2.33	6.1	18.6								
Citrate	1	113	291.4a	±234.77	13.5	1,081.0	74.0	±12	60.0	89.0	294.0	±30	254.0	326.0
	2	112	246.36b	±192.54	0.0	747.4								
Quinate	1	113	30a	±10.44	12.6	72.1	45.0	±0.3	42.0	49.0	21.0	±2	20.0	24.0
	2	111	18.8b	±8.00	0.0	57.4								
Malate	1	113	572.3a	±199.18	177.3	1,102.5	495.0	±64	431.0	582.0	725.0	±46	685.0	789.0
	2	112	490.7b	187.45	38.6	1,276.1								
														

*Data followed by different letters are significantly different (ANOVA followed by Tukey test. p < 0.05).*

A year effect was observed for pH and titratable acidity. In particular, pH differed significantly in every year of analysis according to the Tukey’s test (Table 1), while the total acidity of years 1 and 3 was significantly different from year 2. Furthermore, significant correlations were found among analyzed traits related to fruit flesh acidity. As expected, fruit pH was negatively correlated with titratable acidity in each years of analysis (*r* = −0.27 for year 1, *r* = −0.39 for year 2 and *r* = −0.55 for year 3, with *P* < 0.01) as shown in Table 2.

**TABLE 2:** The Pearson’s correlation coefficients between pH, titratable acidity (TA), citrate, quinate, and malate content calculated on 90 individuals of the ‘Lito’ × ‘BO81604311’ population.

	pH Y1	pH Y2	pH Y3	TA Y1	TA Y2	TA Y3	Malate Y1	Citrate Y1	Quinate Y1	Malate Y2	Citrate Y2	Quinate Y2
pH Year 1	−	−	−	−	−	−	−	−	−	−	−	−
pH Year 2	0.12	−	−	−	−	−	−	−	−	−	−	−
pH Year 3	0.32**	0.52**	−	−	−	−	−	−	−	−	−	−
Titratable acidity Year 1	−0.27*	−0.39**	−0.53**	−	−	−	−	−	−	−	−	−
Titratable acidity Year 2	−0.28**	−0.39**	−0.61**	0.70**	−	−	−	−	−	−	−	−
Titratable acidity Year 3	–0.14	−0.30**	−0.55**	0.60**	0.68**	−	−	−	−	−	−	−
Malate Year 1	–0.14	0.08	0.13	–0.13	−0.21*	−0.34**	−	−	−	−	−	−
Citrate Year 1	–0.15	−0.33**	−0.46**	0.63**	0.60**	0.58**	−0.74**	−	−	−	−	−
Quinate Year 1	0.09	0.27**	0.40**	−0.22*	−0.27*	−0.28**	–0.17	–0.05	−	−	−	−
Malate Year 2	0.09	0.10*	0.21*	−0.29**	–0.21	−0.36**	0.72**	−0.75**	0.01	−	−	−
Citrate Year 2	–0.18	−0.35**	−0.47**	0.57**	0.59**	0.64**	−0.67**	0.87**	–0.13	−0.84**	−	−
Quinate Year 2	0.15	0.22**	0.33**	–0.18	–0.20	–0.16	–0.17	–0.06	0.80**	–0.13	0.05	−

*The correlations between pH and titratable acidity were calculated for the three consecutive years of analysis, while the citrate, quinate, and malate ones only for 1 year.*

The correlation is significant at the 0.05 level (*) and 0.01 level (**).

Regarding the parents, both the malate and citrate were higher in ‘Lito’ than in ‘BO81604311.’ More in detail, in Lito, the malate content was about twice the citrate one (725.0 vs. 294.0 mg/100 g FW) while in ‘BO81604311,’ it is seven times higher (495.0 vs. 74.0 mg/100 g FW). On the contrary, quinate content was double in ‘BO81604311’ in respect to ‘Lito’ (45.0 vs. 21.0 mg/100 g FW).

Regarding the organic acid composition of the whole progeny, malate was predominant in mature fruits (about 64% in both the years), citrate was about half the malate, and quinate was the less abundant (about 3% of the total acidity).

The range of single organic acids contents within the progeny was very wide for all the acids: malate (177.3 up to 1,102.5 mg/100 g FW and 38.6 up to 1,276.1 mg/100 g FW for the first and second year, respectively), citrate (13.5 up to 1,081.0 mg/100 g FW and 0.0 up to 747.4 mg/100 g FW for the first and second year, respectively) and quinate (12.6 up to 72.1 mg/100 g FW and 0.0 up to 57.4 mg/100 g FW for the first and second year, respectively).

No significant correlations were found between malate and pH or titratable acidity in the first year. However, regarding the second year, malate showed a very weak positive correlation with pH (*r* = 0.10, with *P* < 0.05). Citrate, instead, showed strong positive correlations with titratable acidity (*r* = 0.63, and *r* = 0.59, with *P* < 0.01 for the first and second year, respectively) and a moderate negative correlation with pH only in year 2 (*r* = −0.35, with *P* < 0.01). Moreover, malate was very strongly negatively correlated with citrate (*r* = −0.74 and *r* = −0.84, with *P* < 0.01 for the first and second year, respectively). Quinate was found correlated only with pH in the second year (*r* = 0.22, with *P* < 0.01).

### Mapping, Quantitative Trait Loci Analysis, and Physical Map Construction

The published map of ‘Lito’ × ‘BO81604311’ (Dondini et al., 2007) was implemented for improving the coverage of LG8. In particular, two new SNPs (SNP_8G096900 and SNP_8G135400) and two new microsatellite markers (SSR_8G088400 and SSR_8G126800) were identified on four peach gene sequences (Supplementary Table 4).

The new linkage maps coverage was 491.9 cM for ‘Lito’ and 613.9 cM for ‘BO81604311’ with a mean distance among markers of 3.2 and 3.9 cM, respectively. The new LG8 length was 73.2 cM in ‘Lito’ and 37.0 cM in ‘BO81604311.’

The integrated analysis of genotypic and phenotypic data highlighted several major QTL for each acidity trait (Table 3 and Supplementary Figures 1, 2).

**TABLE 3:** Summary of the most significant markers linked to quantitative trait loci (QTLs) of fruit acidity traits by interval mapping (IM) and the Kruskal–Wallis (K) test and percentage of the variance explained for by the QTLs in a F1 apricot progeny of ‘Lito’ × ‘BO81604311’ during the 3 years of analysis.

Trait	Nearest marker	Linkage Group	Position	K	LOD	% Expl.
pH Year 1	udp402	S4	60.1	23.8*******	3.1**	11.9
pH Year 2	udp003	S4	57.2	21.8*******	5.5**	20.9
pH Year 3	eppisf021	S4	52.5	15.1******	5.4**	23.0
Titratable acidity Year 1	BPPCT-009	L6	15.9	7.3***	4.3**	17.4
	PAC003	L7	64.8	11.2*****	3.0*	11.5
	UDAp-470	S8	8.4	11.6*****	3.3**	12.7
Titratable acidity Year 2	UDAp-454	L6	13.6	7.9****	3.0*	12.8
Titratable acidity Year 3	PAC003	L7	64.8	11.8*****	2.7*	12.0
	UDA003	L8	43.9	13.6******	3.1**	13.4
	udp401	S5	59.7	8.6****	2.8*	12
	UDAp-470	S8	8.4	13.2******	4.4**	18.3
Malate Year 1	UDAp-470	L8	25.4	26.6*******	5.9**	21.5
	AMPA111	L8	40.6	28.8*******	7.4**	26.6
	udp409	S8	31.2	34.0*******	8.4**	29.0
Malate Year 2	UDAp-470	L8	25.4	16.9*******	4.3**	16.4
	AMPA111	L8	40.6	24.0*******	6.9**	25.0
	udp409	S8	31.2	32.2*******	8.5**	29.5
Citrate Year 1	CPPCT08	L6	0	14.1******	3.6**	13.7
	UDAp-470	L8	25.4	35.5*******	8.6**	29.6
	AMPA111	L8	40.6	38.1*******	9.0**	31.0
	udp409	S8	31.2	44.1*******	10.8**	35.6
Citrate Year 2	UDAp-454	L6	13.6	10.0****	3.4**	14.5
	UDAp-470	L8	25.4	22.4*******	5.5**	20.2
	AMPA111	L8	40.6	28.3*******	8.0**	28.6
	udp409	S8	31.2	36.1*******	9.3**	32.0
Quinate Year 1	udp401b	L5	47.1	11.6*****	4.1**	15.6
	AMPA-123B	L6	55.1	17.2*******	4.4**	16.8
	AMPA-114	L7	41.1	22.1*******	7.5**	26.9
	UDAp-483	S4	54.2	13.1******	2.8*	11
	AMPA-123B	S6	46.5	16.4*******	5.1**	19.5
Quinate Year 2	BPPCT-041	L5	58.9	3.9**	2.6*	10.9
	udp010	L6	53.1	12.7******	2.9**	11.5
	AMPA-114	L7	41.1	10.8****	3.9**	15.1
	eppisf021	S4	52.5	13.5******	3.5**	14.2

LOD threshold for QTL intervals: *p < 0.05, **p < 0.01.

Kruskal-Wallis significance levels: *p < 0.1, **p < 0.05, ***p < 0.01, ****p < 0.005, *****p < 0.001, ******p < 0.0005, and *******p < 0.0001.

Concerning pH, the analysis identified one region in ‘BO81604311’ associated with this trait in LG4. The QTL showed good stability among years with a LOD score ranging from 3.1 to 5.5 (*P* < 0.01), explaining from the 11.9 to 23.0% of the trait variability in the 3 years (Table 3 and Supplementary Figure 1).

QTLs associated with titratable acidity did not show such good stability among years (Table 3 and Supplementary Figure 1). In ‘Lito,’ QTLs on LG6, LG7 were all present in the first year, while only LG6 in the second year and LG7 and LG8 were observed in the third year. The QTL peak in LG6 is located close to the markers BPPCT-009 and UDAp-454 (explained variability from 12.8 to 17.4%). A QTL in LG8 was found in years 1 and 3 in ‘BO81604311.’ Even in these cases, the LOD score was ranging between 3.3 and 4.4 in ‘BO81604311’ (*P* < 0.01 and explained variability from 12.6 to 18.2%). When present, the QTLs were located almost in the same regions in the different years.

**FIGURE 2:**
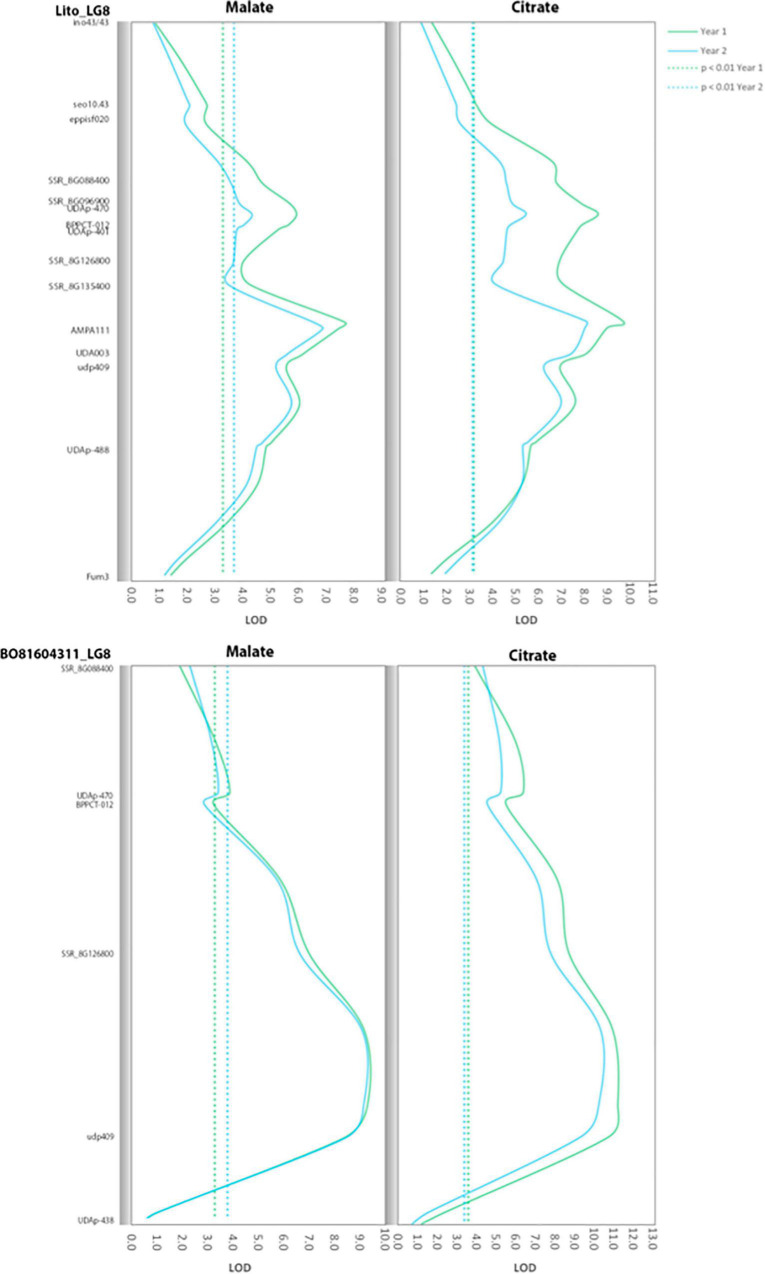
Positions of the quantitative trait loci (QTLs) for citrate and malate contents found in LG8 of ‘Lito’ and ‘BO81604311.’ The dotted line represents the significance threshold of the QTL.

Major QTLs were identified for malate, citrate and quinate in both the parents and years (Figure 2 and Supplementary Figure 2). In the case of malate and citrate QTLs of LG8, peaks were located in a wide region surrounding the SSR marker udp409. In ‘Lito,’ the malate QTL peaks were located close to the marker AMPA111 with LOD values of 7.4 and 6.9 in the 2 years (explained variability of 26.6 and 25.0%, respectively), and of 9.0 and 8.0 for citrate (explained variability of 31 and 28.6%, respectively). In ‘BO81604311,’ QTL peaks were close to udp409 with LOD values of 8.4 and 8.5 for malate (explained variability of 29.0 and 29.6%, respectively) and 10.8 and 9.3 for citrate (explained variability of 35.6 and 32.0%, respectively). The malate and citrate QTLs on LG8 are partially overlapping with the QTL for titratable acidity in the region flanking the UDAp-470 marker (years 1 and 3). As well as for titratable acidity, QTLs for citrate were also found in LG6 located in the region of the marker UDAp-454 (LOD 3.6 and 3.4, respectively, with *P* < 0.01) in ‘Lito’ for the two following years of analysis.

QTLs controlling quinate content were found in ‘Lito’ in LG5, LG6, and LG7 in both years, while in ‘BO81604311’ in LG4, in both the years, and LG6 only in the first year. The QTL on LG6 was present in both parents in the region located close to the AMPA-123B marker (LOD 4.4 and LOD 2.9 in ‘Lito’ and LOD 5.1 in ‘BO81604311’ but only in the first year). However, in ‘Lito’ the strongest QTL for quinate was found in LG7 near AMPA-114 marker in the first year of analysis (LOD 7.5, with *P* < 0.01).

Finally, in order to facilitate the QTL localization, the final ‘Lito’ and ‘BO81604311’ maps were used to determine the physical positions of each marker on the new apricot ‘Stella’ genome sequence (Supplementary Table 5). The location of 187 markers has been successfully blasted on the apricot genome. For 53 SSRs, it was not possible to locate a unique position in the physical map. The order of the ‘Lito’ markers highlighted good collinearity between genetic and physical maps mainly with a few inversions within the same linkage group, tough most of the discrepancies occurred between closest markers. No markers were located on different chromosomes with respect to the genetic map. However, some genomic regions, mainly in LG2 and LG3, showed a very different marker order between genetic and physical maps. The alignment between genetic and physical maps evidenced an overall good coverage of the available maps with a few gaps of more than 5 Mbp (Supplementary Table 5).

### Candidate Genes Identification

**FIGURE 3:**
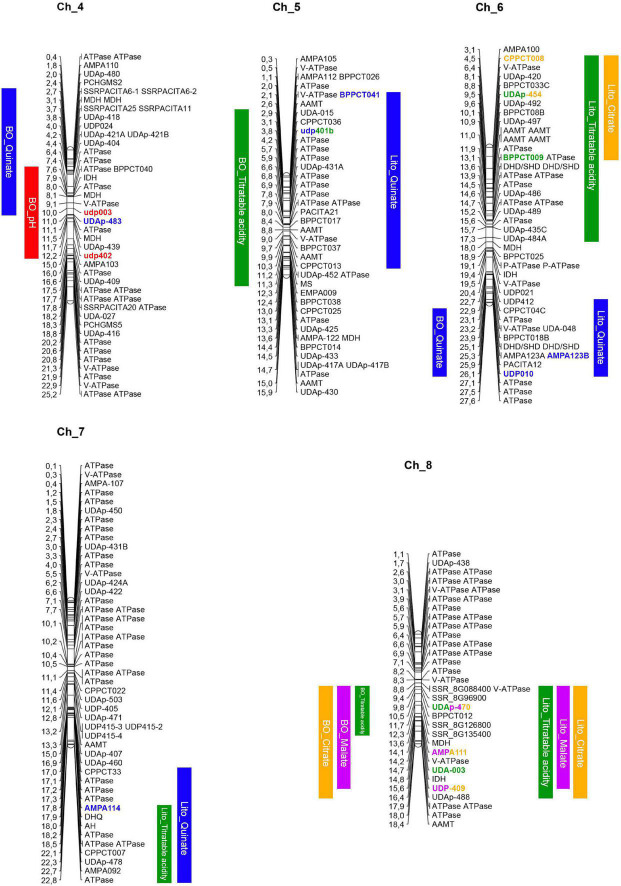
Apricot physical maps including the positions of the QTLs regions (in colored bars for both parents, ‘Lito’ on the right and ‘BO81604311’ on the left) and of the acidity-related candidate genes. IDH, isocitrate dehydrogenase; MDH, malate dehydrogenase; AAMT, aluminum-activated malate transporter; MS, malate synthase; DHD/SDH, bifunctional 3-dehydroquinate dehydratase/shikimate dehydrogenase; DHQ, dehydroquinate synthase.

A total of 263 genes putatively involved in the fruit acidity pathways or in the organic acid compartmentation were found in the apricot genome sequence. The physical position of all the candidate genes is reported in Supplementary Table 6. In most of the QTL regions, at least a candidate gene related to acidity was located (Figure 3).

In details, genes encoding for proteins that take part in malate, citrate, and quinate metabolic pathways have been identified in different QTL regions. Genes encoding for an NAD-dependent malate dehydrogenase (NAD-MDH) and an isocitrate dehydrogenase (IDH) are located near AMPA111 within the QTL for malate and citrate on chromosome 8. In chromosome 6, two genes involved in the quinate metabolic pathway, encoding for a dehydroquinate dehydratase, were identified in regions in which QTLs for titratable acidity and quinate contents, respectively, were located. Furthermore, another NAD-MDH and another IDH were found in the same chromosome close to UDAp-484A flanking a region where the QTL for titratable acidity was located. Moreover, one gene for 3-dehydroquinate synthase (DHQ) was also found within the QTL for quinate and titratable acidity on chromosome 7. In addition, an isoform of the aconitate hydratase (AH) was found in the same region. Moreover, two cytoplasmic NAD-MDH genes and one IDH were located in a QTL region for both pH and quinate content on chromosome 4. In the latter QTL, other two NAD-MDH genes were found. In chromosome 5, a gene encoding for a malate synthase (MS) was found within a QTL region for titratable acidity.

Regarding putative membrane transporter genes, three aluminum-activated malate transporter (AAMT) were located on chromosome 6 (PruarS.6G181900, PruarS.6G182300, and PruarS.6G182700) within a QTL for titratable acidity of years 1 and 2 while the other two isoforms in a QTL region for titratable acidity and quinate content in chromosome 5.

Finally, four ATPases, one of which is vacuolar (V-ATPase), were found in chromosome 4 in a QTL region for pH. Another V-ATPase was found in chromosome 8, in the region associated with titratable acidity, malate, and citrate contents, and one H-ATPase also related to titratable acidity was identified at the end of chromosome 7.

## Discussion

Significant differences between pH and TA values were observed in different years of sampling and this could be probably because of the yearly effects of the climatic condition variability, in agreement with literature data which reports the interaction of environmental conditions and acid content in the fruit of various species either due to water availability (Mills et al., 1996; Veit-Köhler et al., 1999; Hudina and Śtampar, 2000; des Gachons et al., 2005; Orts et al., 2005; Pérez-Pastor et al., 2007; Kallsen et al., 2011; Bartolini et al., 2015) or temperatures (Ruffner et al., 1983; Wang and Camp, 2000; Gautier et al., 2005).

In general, the fruit acidity traits showed transgressive distributions with values higher or lower than parents in several cases. The genetic background of the parents is known to influence the segregation of acidity-related traits as previously reported by several authors (Salazar et al., 2013; García-Gómez et al., 2019).

Citrate and quinate contents did not show a normal distribution in our progeny in both the years of phenotyping, probably due to the presence of a major gene that could explain high-trait variability (Alonso-Blanco et al., 2006).

Malate is reported as the major organic acid in apricot fruits (Bassi et al., 1996). However, some individuals of the analyzed progeny showed higher citrate than malate contents, but it is known that organic acid contents in apricot fruits are largely dependent on the genotype (Gurrieri et al., 2000; Bureau et al., 2001). Citrate and malate contents are metabolically related traits as they are Krebs cycle intermediates (Famiani et al., 2015). These two traits resulted negatively correlated in our progeny as reported by other authors but on an apricot germplasm collection (Baccichet et al., 2022).

The few markers added on the LG8 of both the parental maps resulted in the improvement of the coverage in regions where fruit acidity QTLs are located. The positions of the new markers on LG8 were in agreement with those observed in the peach genome thus confirming the synteny between the peach and apricot genomes, already described in the literature (Dondini et al., 2007; Jung et al., 2009). To date, this analysis is strongly facilitated by the release of the apricot genome (Groppi et al., 2021).

Most of the mapped ‘Lito’ SSRs have been identified in the Stella genomic sequence. The few SSRs for which the genomic location was not found were mainly developed from other *Prunus* species. Therefore, possible differences within the primer sequences between the two species may have hampered their search in the apricot genome. The few discrepancies observed in the alignment between the ‘Lito’ genetic map and the Stella physical map could be due to different reasons, i.e., genotyping or assembly errors or because of their different genetic background. To this extent, ‘Lito’ and ‘Stella’ are sharing the Sharka resistance region on chromosome 1 (De Mori et al., 2019), but after genetic diversity analysis, Stella clustered very far from both ‘Stark Early Orange’ and ‘Tirynthos,’ the ‘Lito’ parents (Geuna et al., 2008).

The main acidity-related QTLs in our progeny have been found in LGs 4, 5, 6, 7, and 8 while no QTLs have been identified in LGs 1, 2 and 3. The QTL positions of the traits showed rather good stability over years thus confirming the robustness of the analyses. QTLs for titratable acidity are generally in agreement with a preliminary work by Ruiz et al. (2010) that reported QTLs for titratable acidity in LG6, LG7 and LG8 on the same population.

Titratable acidity is a polygenic trait (Signoret et al., 2004; Ruiz and Egea, 2008; Ruiz et al., 2010; Salazar et al., 2013, 2014, 2016) and several QTLs for titratable acidity have been found in LGs 6, 7 in ‘Lito’ and LG8 in ‘BO81604311.’ Salazar et al. (2013) described the presence of titratable acidity QTLs in LG1, LG2, and LG4 while García-Gómez et al. (2019) identified QTLs for this trait in LG2 and LG8. However, titratable acidity involves several genes organized in complex biosynthetic pathways and the different genetic backgrounds can highlight variability for this trait from different genomic regions. Citrate and malate are Krebs cycle intermediates while quinate is mainly synthesized by the shikimate pathway (Famiani et al., 2015).

Concerning pH, the QTL in LG4 of ‘BO81604311’ was also reported in the same position by Salazar et al. (2013). No QTLs were found in ‘Lito’ suggesting that the segregation of fruit pH in this progeny is mostly dependent on a region in LG4 of ‘BO81604311.’

To the best of our knowledge, no previous studies reported the QTLs for single organic acids by GC in apricot. Most of the QTLs for titratable acidity and individual organic acids are overlapping for their location within the genome but not perfectly. Some of the QTLs were identified only by using GC data and not by titratable acidity. In particular, the ‘Lito’ QTLs for malate and citrate of LG8, and also the QTL for quinate on LG5, were not revealed by the analysis of titratable acidity data. Analogously, the QTL on LG6 of ‘BO81604311’ was found only by using the quinate data. Furthermore, a shift of the QTL peak along the LG8 of ‘BO81604311’ was observed comparing the QTL curves obtained by the analysis of the malate and titratable acidity datasets (the QTL shifted its position of about 30 cM from udp409 to UDAp-470). Similarly, a shift has been observed also between the QTLs for quinate and titratable acidity on LG 6 and LG7 of ‘Lito.’ Finally, the QTLs identified by using the GC data showed a higher magnitude in respect to the corresponding QTLs identified with titratable acidity (i.e., the LOD score for the malate QTL peak of ‘BO81604311’ on LG8 is 8.42 while for titratable acidity was only 4.45). This result was expected since titratable acidity is the result of all the organic acids present in the fruit and a confounding effect due to the different components has to be considered.

Candidate genes for organic acid synthesis, degradation, transport, and compartmentation have been found in several QTL regions. Concerning malate, several authors agree on the importance of NAD-malate dehydrogenases that catalyses the reversible conversion of oxaloacetate into malate (Sweetman et al., 2009; Yao et al., 2009). Significantly, a member of this gene family (PruarS.8G247000) was found within the QTL region for malate with a high LOD score (on LG8 of ‘Lito’). The same goes for the isocitrate dehydrogenase, another key enzyme in the TCA, found in chromosome 8 inside the QTL for citrate. These two genes are robust candidates for the control of organic acid in the fruit flesh; their presence in the same region and their common metabolic pathway can also explain the co-localization of their QTLs.

Interestingly, the analysis of candidate genes in QTL regions suggests that accumulation of citrate and quinate in the analyzed progeny might depend predominantly on enzymes involved in their metabolism, rather than on their accumulation into vacuoles. Additional studies will be needed to confirm this hypothesis, which, however, seems consistent with previous studies reporting the crucial role of citrate metabolism instead of vacuolar accumulation in peach (Zheng et al., 2021).

Nonetheless, vacuolar storage indeed plays a fundamental role in organic acid accumulations. The putative aluminum-activated malate transporter found in LG6 and associated with titratable acidity could be part of the family already described in *Arabidopsis thaliana* (Kovermann et al., 2007; Meyer et al., 2011), grape (Rongala, 2008), and apple (Bai et al., 2012) and can be responsible for the transport of malate through the tonoplast. In addition, these genes were also already identified in apricot (Groppi et al., 2021).

Finally, the ATPase genes found in LG4 can be linked to pH and might thus be underlying the observed QTL effect on this trait. The V-ATPase activity leads to the increase of H^+^ into the vacuole and contributes to the “acid trap” mechanism that allows the increment of the vacuolar organic acids concentration. This could also explain the presence of these genes in regions related to titratable acidity QTLs as in LG6 and LG8.

## Conclusion

In this study, the identification of several QTLs related to apricot flesh fruit acidity traits is reported: pH, titratable acidity, malate, citrate and quinate content. A strong influence of the year on the variability observed for each trait has been found and which also impacts the QTLs location. In particular, QTL is linked to pH (LG4), titratable acidity (LG6, LG7, and LG8), malate and citrate (LG8) and quinate (LG5, LG6, and LG7) were identified. All the markers related to those traits can, thus, help in future breeding programs of apricot by improving the marker-assisted selection of this species.

Moreover, the release of the new apricot ‘Stella’ genome enables the construction of the physical map of ‘Lito.’ Furthermore, several genes involved in the synthesis and degradation of the compounds forming acidity and vacuolar transporter have been identified and located in the physical map and some co-localize with acidity-related QTLs. These genes can be subjected to further studies in the future to assess their effective role in the determination of apricot fruit acidity.

## Data Availability Statement

The original contributions presented in the study are included in the article/Supplementary Material, further inquiries can be directed to the corresponding author.

## Author Contributions

LD and ST conceived and designed the experiments. DB, SF, and ML contributed to plant materials. ST, LD, PD, MA, CC, FG, CD, and SA contributed to molecular analyses. LD, ST, PD, CD, SA, LB, and YD carried out the statistical analyses. CD, ST, LD, and LB wrote the manuscript. LD, ST, PD, YD, and CD critically revised the manuscript. All authors performed the field experiments and fruit quality analyses, and read and approved the final version of the manuscript.

## Author Disclaimer

The views expressed in this study are the sole responsibility of the authors and do not necessarily reflect the views of the European Commission.

## Conflict of Interest

The authors declare that the research was conducted in the absence of any commercial or financial relationships that could be construed as a potential conflict of interest.

## Publisher’s Note

All claims expressed in this article are solely those of the authors and do not necessarily represent those of their affiliated organizations, or those of the publisher, the editors and the reviewers. Any product that may be evaluated in this article, or claim that may be made by its manufacturer, is not guaranteed or endorsed by the publisher.

## Footnotes

1
http://www.fao.org/faostat/en

2
https://cran.r-project.org/web/packages/agricolae/index.html

3
https://cran.r-project.org/web/packages/multcomp/index.html

4
https://www.rosaceae.org/

5
https://www.rosaceae.org/search/genes

## Supplementary Material

Supplementary Figure 1 Charts obtained from the QTL analysis of ‘Lito’ and ‘BO81604311’ for quinate content, titratable acidity, and pH. The dotted line represents the significance threshold of the QTL.

Supplementary Table 1 Primer sequences of the four new markers added to the ‘L × B’ map. For single nucleotide polymorphism (SNP) markers, also the allele-specific primer is reported as well as amplicon length.

Supplementary Table 2 Polymerase chain reaction amplification protocols for the new SNP markers.

Supplementary Table 3 The Shapiro–Wilk normality test results (W) and *p*-values significances (*).

Supplementary Table 4 New SSR and SNP marker positions in the peach (*Prunus persica*) genome.

Supplementary Table 5 Alignment of the genetic and physical maps of ‘Lito.’ The position of markers is expressed in cM and in bp for each of the ‘Stella’ chromosomes. The list of markers that were not found in the ‘Stella’ genome sequence is also reported.

Supplementary Table 6 Names, genomic location, and predicted function of the acidity-related candidate genes found in the ‘Stella’ apricot gene database. The quantitative trait loci (QTLs) associated with each gene is also reported.
